# Workload regulation by Sudarshan Kriya: an EEG and ECG perspective

**DOI:** 10.1007/s40708-016-0055-1

**Published:** 2016-07-18

**Authors:** Sushil Chandra, Greeshma Sharma, Mansi Sharma, Devendra Jha, Alok Pakash Mittal

**Affiliations:** 1 Institute of Nuclear Medicine and Allied Science (INMAS), Defence research and development organization (DRDO), Delhi, India; 2 Thapar University, Patiala, Punjab India; 3 Scientific Analysis Group (SAG), Defence research and development organization (DRDO), Delhi, India; 4 Netaji Subhas Institute of Technology (NSIT), Dwarka, Delhi, India

**Keywords:** Workload, Sudarshan Kriya Yoga (SKY), Physiological signals, Stress

## Abstract

Sudarshan Kriya Yoga (SKY) is a type of rhythmic breathing activity, trivially a form of Pranayama that stimulates physical, mental, emotional, and social well-being. The objective of the present work is to verify the effect of meditation in optimizing task efficiency and regulating stress. It builds on to quantitatively answer if SKY will increase workload tolerance for divided attention tasks in the people sank in it. EEG and ECG recordings were taken from a total of twenty-five subjects who had volunteered for the experiment. Subjects were randomly assigned to two groups of ‘control’ and ‘experimental.’ Their objective scores were collected from the experiment based on NASA’s multi-attribute task battery II and was utilized for workload assessment. Both the groups had no prior experience of SKY. The experimental group was provided with an intervention of SKY for a duration of 30 min everyday. Pre- and post-meditation data were acquired from both groups over a period of 30 and 90 days. It was observed that subjective score of workload (WL) was significantly reduced in the experimental group and performance of the subject increased in terms of task performance. Another astute observation included a considerable increase and decrease in the alpha and beta energies and root mean square of the EEG signal for the experimental group and control group, respectively. In addition to this sympathovagal balance index also decreased in experimental group which indicated reduction in stress. SKY had an effect on stress regulation which in turn enhanced their WL tolerance capacity for a particular multitask activity.

## Introduction

SKY is a rhythmic breathing activity consisting of the five stages i.e., Ujjayi, Bhastrika, Om, Sudarshan kriya, and Yoga Nindra [1–3]. Sudarshan kriya is a Sanskrit word which literally means ‘proper vision by purifying action.’ It is an advanced form of rhythmic, cyclic breathing with slow, medium, and fast cycles. SKY is a practice of wellness [4] which reduces stress, produces relaxation, and increases sensitivity in sensory transmission, leading to an increase in attention and vigilance [2, 5]. As the levels of both psychological and physiological stress are tuned up and down, a non-linear effect on workload is evident [6]. This is a day-to-day phenomena so the productivity can be rigorously enhanced by practicing SKY on a daily basis. MATB-II, the task designed for assessing the multitasking attributes, was used for workload assessment at two levels: low workload (LWL) and high workload (HWL) [7]. Increased task difficulty (LWL vs. HWL) is associated with a characteristic pattern of electroencephalographic (EEG) activity: increased power in the beta bandwidth, increased theta activity at frontal sites, and suppression of alpha activity [8–12]. Since gamma energy increases when a person gets engaged in a higher level of mental task or there is a voluntary allocation of attention, it is quite evident that there is an increase when the person is involved in a switching task [13–15]. Engagement index is correlated with workload level and gives a quantitative assessment of EEG index for workload [16–18]. On the other hand, mental workload level can be interpreted from electrocardiography (ECG) signals mainly by heart rate variability (HRV) component [19, 20]. HRV increases with an increase in mental effort because workload has a direct effect on vagal tone [21]. The objective of this work is to find out the effect of SKY technique on individuals subjected to different levels of workload perfused using MATB-II. Physiological signals are cross-validated with objective and subjective scoring of individuals. SKY has proven effects in relieving stress, but it has been a prolific challenge to know that SKY can be beneficial in improving performance thereby enhancing workload capacity. Additionally, it is also important to dig out whether there is any effect on physiological signals at different workload levels or does it also have a practice effect? The control group is chosen to remove such biases. SKY has a qualitative effect on physiological signals, but it can be quantified based on objective and subjective scoring. The conclusive result obtained from EI features is further routed through SVM and NN classifiers separately to amplify their significance. The paper is organized as follows. Section 2 presents a description of the experimental design and demographic details of participants, and also briefly describes the extracted features and classifiers that were used. Section 3 describes results. A detailed review of the literature related to the physiological signals and workload for SKY practitioner is given in Sect. 4, which also discusses the results obtained in this work. Finally, conclusions are given in Sect. 5.

## Materials and methods

### Participants

Subjects were taken from the border road organization with no ongoing or recent mental health problems or neurological disorders. All subjects were naive to SKY with no normal visual acuity and were administrated under art of living (AOL) group. AOL is a non-governmental organization, which is involved in humanitarian projects with a special focus on stress elimination. After taking permission from all 25 participants (Male, average age = 40 year), 10 control and 15 experimental subjects were exposed to the experimental protocol. Participants were randomly categorized in the both groups and matched on age and education. The control group was not given SKY sessions.

### Experimental procedure

A consent form is filled prior to experiment and groups were categorized randomly for minimizing any biases. Prior to SKY, both groups underwent practice session of MATB-II for 5 min. Task and duration of task presentation were customized in MATB-II for LWL and HWL after conducting small pilot study on ten subjects. Duration was selected from 2 to 20 min for each trial consisted of 2 min in succession. We found that best suitable time was 8 min for assessing workload and subsequently its classification. Data (pre) were collected for both groups using physiological signals i.e., EEG and ECG, and MATB task was given for 5 min for baseline (BL) recording and 8 min for each level, LWL and HWL (C1 BL, C1 LWL, C1 HWL, E1 BL, E1 LWL, E1 HWL for pre-control baseline, low workload, high workload, pre-experimental baseline, low workload and high workload, respectively). Only experimental group was exposed to SKY technique in a workshop of 7 days. After that they pursued SKY technique under experimenter’s observation for 90 days. Post-recording was done after 90 days for both groups (C2 BL, C2 LWL, C2 HWL, E2 BL, E2 LWL, E2 HWL) to notice longitudinal effect of meditation. Post-recording included similar baseline and MATB task for 8 min for LWL and HWL, respectively; simultaneously, EEG and ECG data were acquired during the task. All experimental procedures were approved by the ethical committee at INMAS, DRDO.

### Instruments

Multiple attribute task battery (MATB-II): MATB is designed for multitasking that can evaluate workload indirectly. It consists of a two-dimensional tracking, system monitoring, communication and resource management tasks. Resource management task is to maintain the fluid volume in tanks A and B in the indicated ranges operating eight pumps. System monitoring is maintaining the normal state of lights that is green is ON and red is OFF and four scales that is the level has to be around the center. Score depends on the response time. Tracking is keeping the target at the grid center in manual mode while in autopilot mode no action is required. RMSD from center in pixel point gives the scores that lie in the range of 0 to 300. Communication task is writing radio frequency received as an audio message. MATB operates in two modes namely train mode to familiarize the subjects with tasks and test mode that includes varying the task difficulty in fixed time interval. Workload Rating Scale (WRS): It is the subjective assessment of workload during task. The subscales are Mental Demand, Physical Demand, Temporal Demand, Own Performance, Effort, and Frustration. Physiological Signals Recording: EEG signals were acquired by using a 14 channel acquisition system [19], according to the 10–20 international system of electrodes. Reference electrode was kept at right and the left mastoids. It was sampled at a rate of 128 Hz, and discretized using a 12-bit A/D resolution. Input impedance was kept to under 5 KΩ. The data were filtered using a 4th order Butterworth bandpass filter with a 24 dB/octave configuration. After that, power line interferences were removed using a 50 Hz notch filter. The ECG data were acquired using a 4-channel MP150 system, sampled at a rate of 500 Hz. The signals were acquired according to the basic Einthoven triangle paradigm. The data were discretized by using a 16-bit A/D converter.

### Signal analysis

The wavelet transform (WT) is another alternative for time frequency analysis. Un-like the short time Fourier transform (STFT), the time frequency kernel for the WT-based method can better localize the signal components in time frequency space. This efficiently exploits the dependency between time and frequency components. Morlets wavelet, which predominantly is localized by a Gaussian window, has the inherent advantage of providing a good synchrony between spatial and frequency resolution. Hence, the discrete wavelet transform (DWT) can be accordingly derived empirically along these lines. Discretization of WT can be carried out with some unpretentious deliberations on the adjustment of the wavelet design by dilation. It is quite logical that the Nyquist criterion must be obliged since no initial condition of band-limiting has been put in. The number of elements for a scale can be reduced if the frequency bandwidth is also reduced. This requires a band-limited wavelet. The decomposition provides a very informative illustration of the reduction of elements. This decomposition is established on a stage wise dichotomy of the frequency band. The search for a discrete transform that is well localized in both spaces leads to a multiresolution analysis.

### Spectral features of EEG signals

#### Energy

Energy of a signal can be quantified as the summation of all energies, corresponding to the different frequency resolution levels of the signal. The signal is segregated at different resolution levels using the WTs. It is calculated for beta, alpha, and gamma band with the following formulae: energy $E_{q}^{p}$ of a spectral frequency band and total energy *E*_*tot*_ can be defined as

1 $$E_{q}^{p} = \sum\limits_{{ - \infty }}^{\infty } {f_{q}^{p} (t^{2} ) = E_{t} } ,$$

2 $$E_{{tot}} = ~\mathop \sum \nolimits^{{}} p = ~1^{{2p}} E_{q}^{p} ,\,$$

Normalized value is given by

$$Pl = \frac{{E_{l} }}{{E_{{tot}} }}.$$

#### Root mean square (RMS)

It gives a measure of the spread of a set of data and in case of signals, it quantifies power from the overall amplitudes obtained in the EEG time series. The RMS for a collection of N values $x_{{\text{1}}} ,x_{{\text{2}}} , \ldots x_{n}$ is

3 $$x_{{rms}} = \sqrt {\frac{1}{N}\sum\limits_{{i\; = \;1}}^{N} {x_{i}^{2} } } = \sqrt {\frac{{x_{1}^{2} + x_{2}^{2} + \cdots + x_{N}^{2} }}{N}}$$

Low RMS value indicates completely desynchronized activity [1].

#### Engagement index

EEG engagement index is defined as

$$\frac{{\beta _{{{\text{psd}}}} }}{{\alpha _{{{\text{psd}}}} + \theta _{{{\text{psd}}}} }},$$

where *β*_psd_, *α*_psd_, and *θ*_psd_ are power spectral density of alpha, beta, and theta bands, respectively [34]. The estimated PSD provides some precise information about the structure of the stochastic process. In this study, Welch method is used for PSD extraction. In the Welch method for calculating PSD, data are first segmented and then windowed prior to calculating PSD of the signal. The modified periodogram is given by

4 $$I_{L}^{i} (\omega ) = \frac{1}{{LU}}\sum\limits_{{m\; = \;0}}^{{L - 1}} {\left| {x_{i} (n) \times \omega (n) \times e^{{ - j\omega n}} } \right|^{2} } ,$$

where *L* is the length of the signal and *U* is the normalization factor.

5 $$P_{{xx}}^{\omega } = ~\frac{1}{m}\sum\limits_{{i\; = \;0}}^{{M - 1}} {I_{L}^{i} } (\omega ),$$

where $P_{{xx}}^{\omega }$ is the welch power spectrum. M is the number of data segments.

### ECG features

One of the most generic attributes of the cardiac sequence used when empirically calculating the heart period is the R-wave peak. This is due to the fact that the R wave is expressed as a strident positive peak trailed by a negative ricochet in the ECG waveform. This is one of the reasons why the R-wave is so easily distinguishable and unique. Pan-Tompkins algorithm [33] is the most illustrious algorithm used for detecting the QRS complex. Beat-to-beat variation in the R–R interval is quantified as the HRV. The HRV analysis can be done both in the time domain as well as frequency domain. In an ECG recording, each QRS complex is detected and then the instantaneous heart rate is determined (which, apparently is known as the normal–normal ratio). NN50 signifies the number of braces of contiguous R–R intervals. pNN50 corresponds to the actual NN50 count divided by the summation of the RR intervals. The RR time interval can be calculated as follows:

6 $$T = \frac{{x_{n} - x_{{n - 1}} }}{{F_{s} }},$$

where T is the time and *F*_*s*_ is the sampling frequency. Three widely used components can be found in HRV power spectrum: LF (0.04–0.15 Hz): a low frequency component that is mediated by both the sympathetic nervous system (SNS) and parasympathetic nervous system (PNS); HF (0.15–0.4 Hz): a high frequency component mediated by the PNS; and LF/HF: LF to HF ratio that is used as an index of autonomic balance. This ratio is also termed as sympathovagal balance index (SVI) [22].

### Classifiers

#### Support vector machines

Support vector machine (SVM) is supervised learning classifier which conceptually implements the following idea: input vectors are non-linearly mapped to a very high dimension feature space. A linear decision surface is evangelized by taking this feature space into consideration. Given a training set of instance-label pairs *x*_*i*_, *y*_*i*_, *i* = 1, 2, …*k* where *x*_*i*_ ∈ *R*_*n*,_ *y*_*i*_ ∈ i{−1,1}, SVM essentially solves the following optimization problem

7 $$\min \nolimits_{{w,b,\varepsilon }} \frac{1}{2}w^{T} w + C\mathop \sum \limits_{{i = 1}}^{k} \varepsilon _{i} ,$$

subject to

8 $$y_{i} (w^{T} \phi (x_{i} ) = b) \ge 1 - \in _{i} ,$$

where *ϵ*_*i*_ ≥ 0. Here training vectors *x*_*i*_ are mapped into a higher dimensional space by the function $\phi$. Then SVM finds a linear separating hyperplane with the maximal margin in this higher dimensional space. The hyperplane is constructed as a set of weights *w*, data points *x* and bias *b*, such that

9 $${\text{w}}.{\text{x }} + {\text{ b }} = {\text{ }}0.{\text{ }}$$

The kernel function in SVM maps the training data into kernel space. For selection of the kernel function, a Gaussian Radial Bias kernel function is used as it gives good accuracy in handling large EEG data over other kernels. The samples can be mapped in a non-linear fashion, which is another attribute of this function. The corresponding SVM is known as RBF SVM. This function is mathematically expressed as follows.

10 $$k(x,x^{\prime } ) = \exp \left( {\frac{{ - \left\| {x - x^{\prime } } \right\|^{2} }}{{2^{2} }}} \right)$$

#### Artificial neural networks

An artificial neural network can be demarcated as a mathematical exemplification of a regular phenomenon. The artificial neurons can be connected in a very structured and uniform way so that they accurately represent the attributes of a system. There have been some standardized designs of ANNs developed by scientists which has been used for many applications all around the world. ANNs have redefined science in a much simpler way. By taking the generalized form of Widrow-Hoff learning rule into consideration, the error back-propagation algorithm was used [23]. The back-propagation algorithm is actually based on the gradient descent algorithm, where initially the performance function is taken into consideration and then the network weights are moved accordingly (through the negative gradient). Lots of variations are possible in this algorithm. Well-trained error back-propagation algorithms give reasonably good answers, even when they have been presented with inputs which have not come under their peripherals before.

## Results

**Fig. 1:**
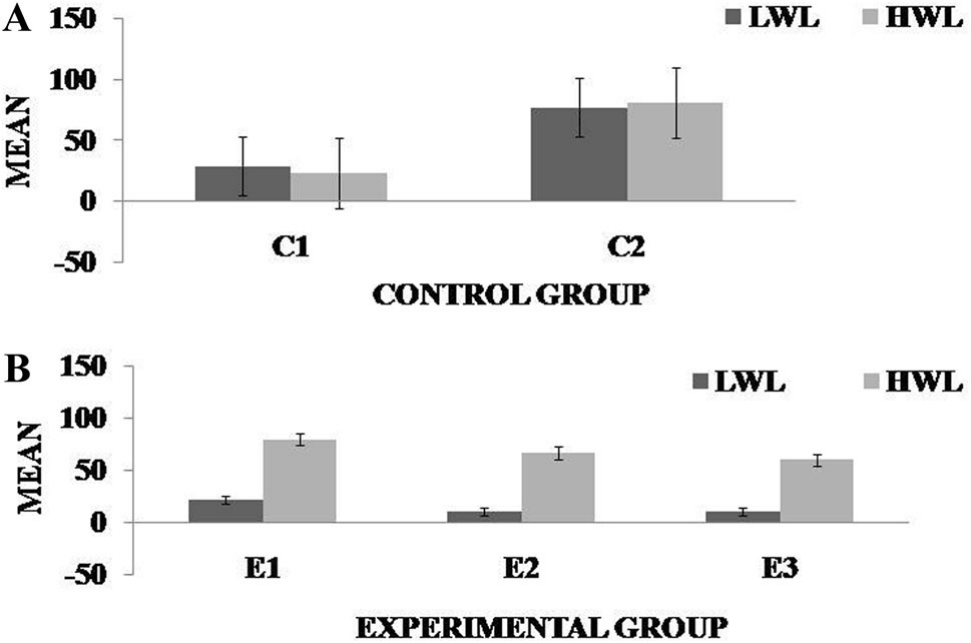
Mean subjective score for low workload level (LWL) and high workload level (HWL) with standard error as *error bar* (±1SE) **a** and **b** for control and experimental groups, respectively. *C1* Pre-control group at 0-day time period, *C2* post-control group after 30 days period, *E1* pre-experimental group prior exposure to SKY, *E2* post-experimental group after 30 days period, *E3* post-experimental group after 90 days period. 0-day time period was defined as the time period prior to experiment beginning

**Fig. 2:**
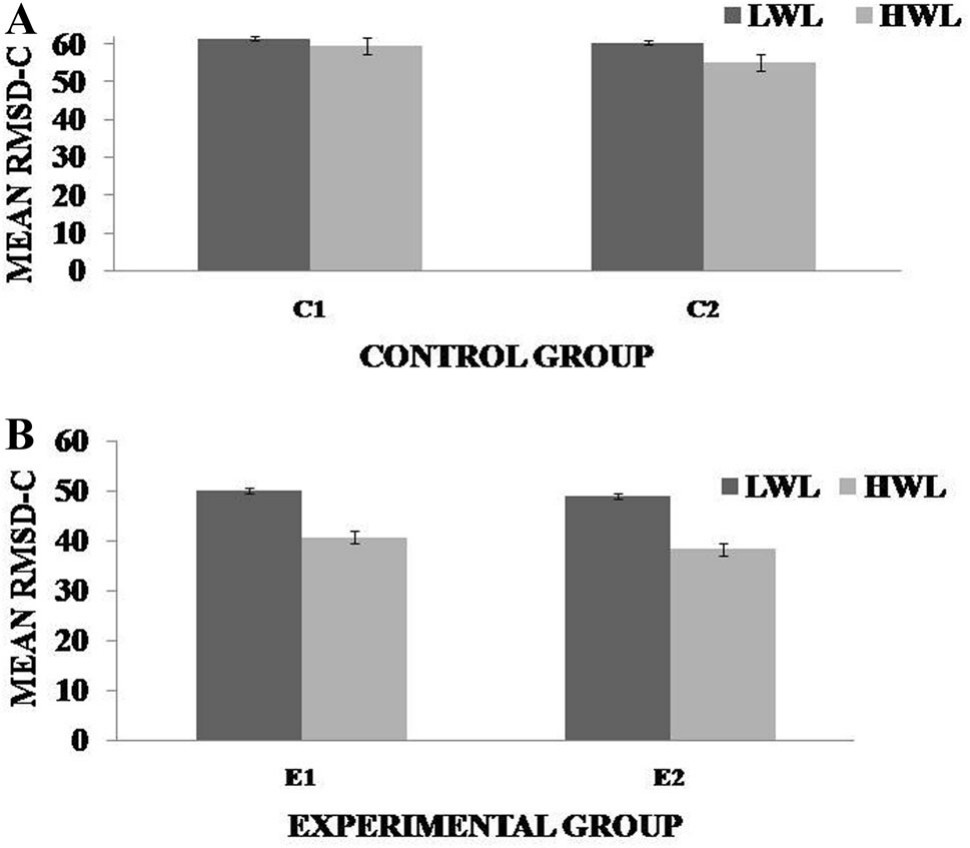
Mean root mean square deviation (RMSD) for low workload level (LWL) and high workload level (HWL) with standard error as error bar **a** and **b** for control and experimental groups, respectively. It is deviation from center point in pixel units. *C1* Pre-control group at 0-day time period, *C2* post-control group after 30 days period, *E1* pre-experimental group prior exposure to SKY, *E2* post-experimental group after 30 days period

**Fig. 3:**
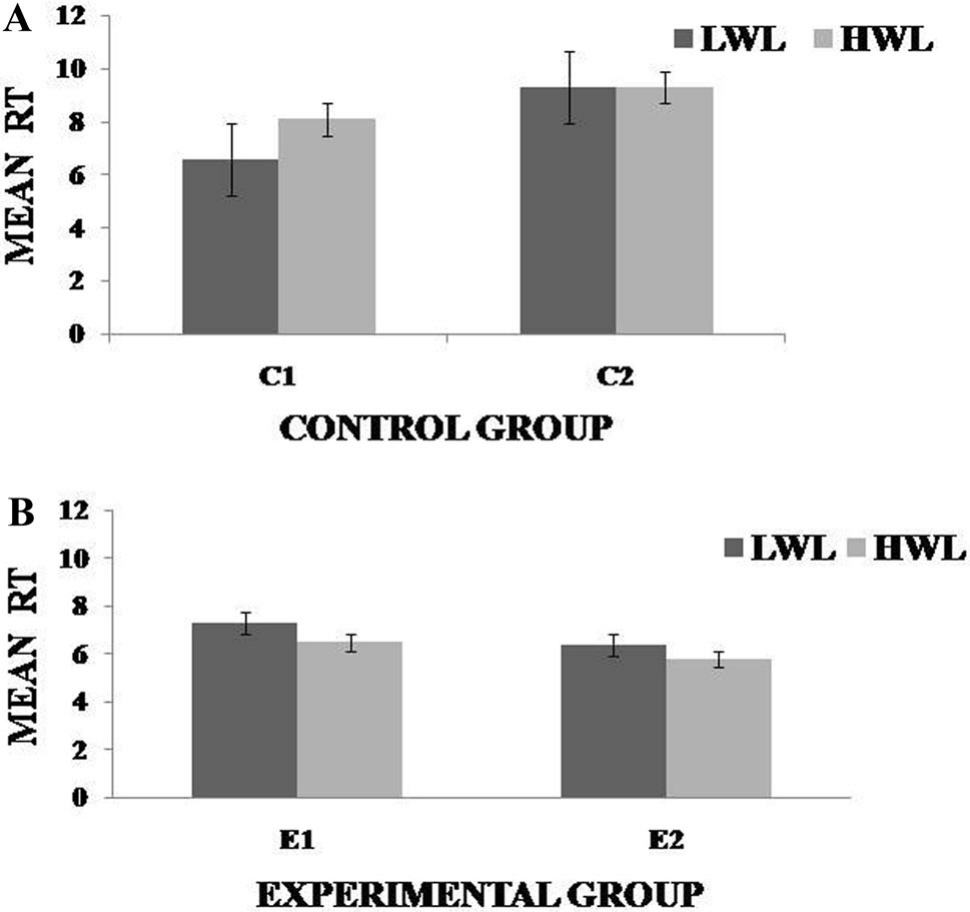
Mean response time (RT) for low workload level (LWL) and high workload level (HWL) with standard error as *error bar* **a** and **b** for control and experimental groups, respectively. *C1* Pre-control group at 0-day time period, *C2* post-control group after 30 days period, *E1* pre-experimental group prior exposure to SKY, *E2* post-experimental group after 30 days period

**Fig. 4:**
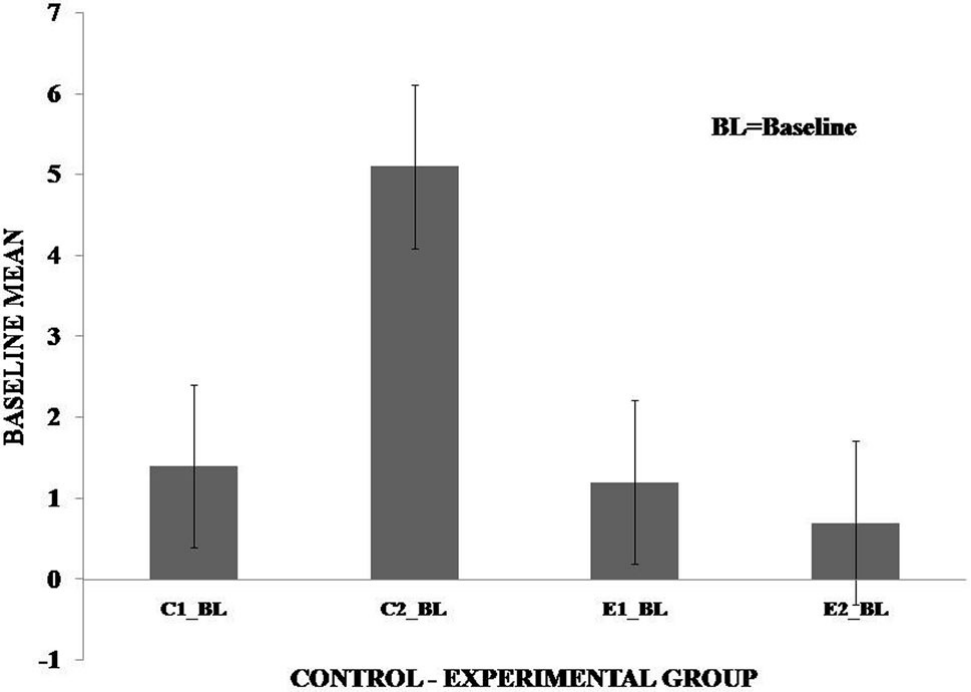
Mean value of sympathovagal balance index (SVI) for baseline (BL) condition in Experiment with standard error as *error bar*. Data were compared among groups for the time periods of 0 and 30 days. *C1 _BL* Pre-control group at 0-day time period, *C2 _BL* post-control group after 30 days period, *E1 _BL* pre-experimental group prior exposure to SKY, *E2_BL* post-experimental group after 30 days period

**Fig. 5:**
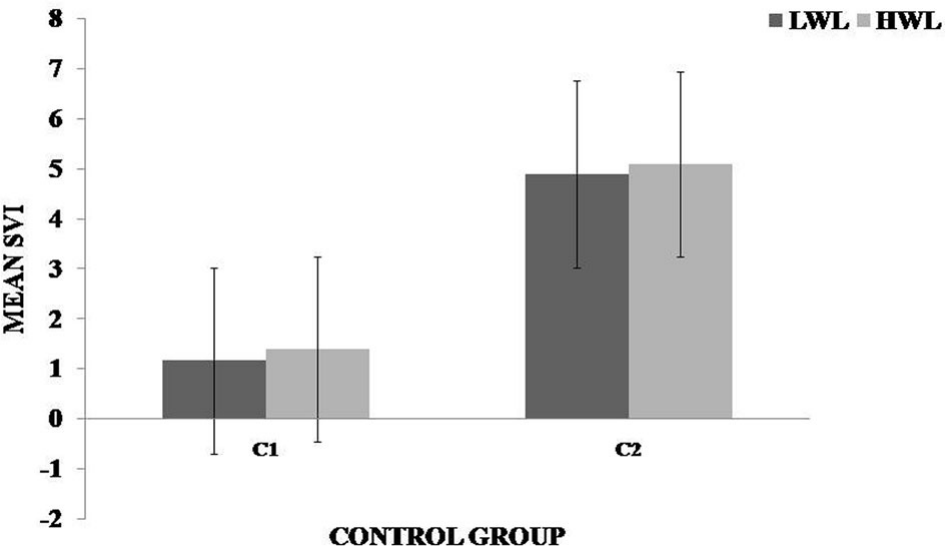
Mean value of sympathovagal balance index (SVI) for low workload (LWL) and high workload (HWL) condition in experiment with standard error as *error bar*. Data were compared within the control group for the time periods of 0 and 30 days. *C1* Pre-control group at 0-day time period, *C2* post-control group after 30 days period

**Fig. 6:**
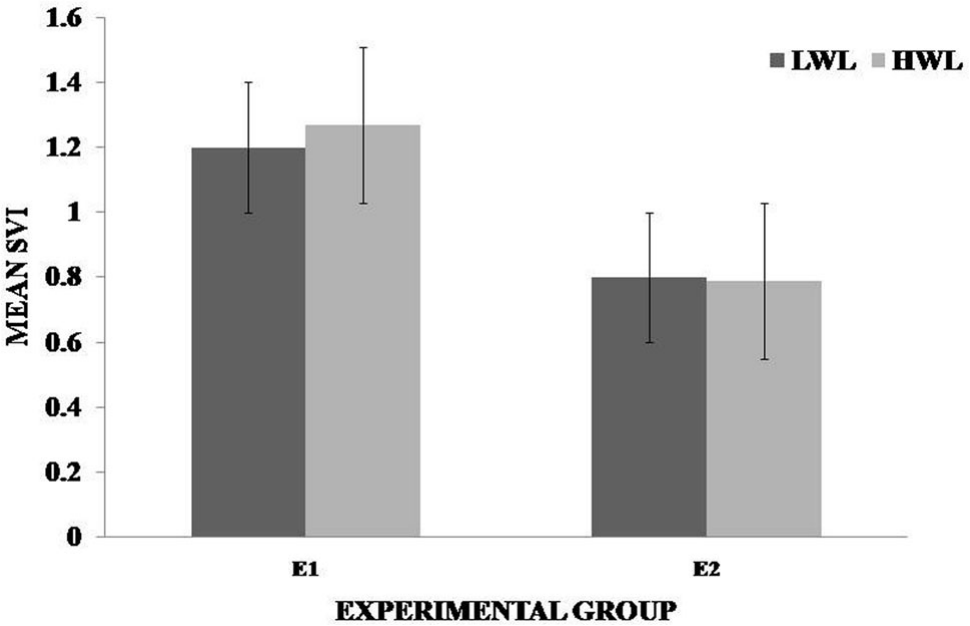
Mean value of sympathovagal balance index (SVI) for low workload (LWL) and high workload (HWL) condition in experiment with standard error as *error bar*. Data were compared within the control group for the time periods of 0 and 30 days. *E1* pre-experimental group prior exposure to SKY, *E2* post-experimental group after 30 days period

**Fig. 7:**
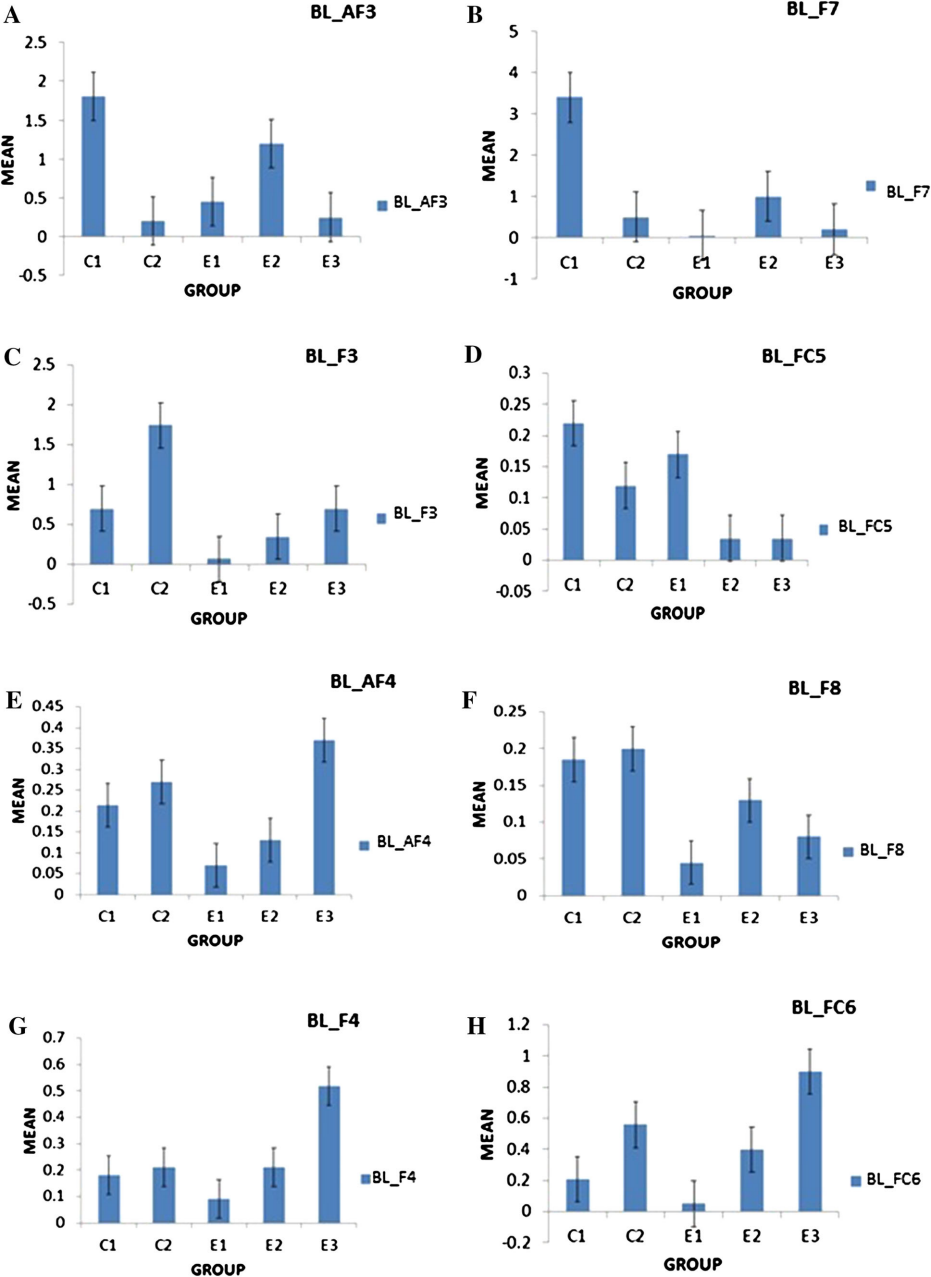
Alpha energy (4–8 Hz) average plotted for baseline (BL) condition between groups as to demonstrate energy distribution between pre- and post-conditions to highlight effect of SKY with standard error as *error bar*. Units were displayed as a magnitude of 1*100000. *BL_AF3, BL_F7, BL_F3, BL_FC5, BL_AF4, BL_F8, BL_F4, BL_FC6* represented mean energy variance for baseline condition for EEG channels AF3, F7, F3, FC5, AF4, F8, F4, FC6, respectively

**Fig. 8:**
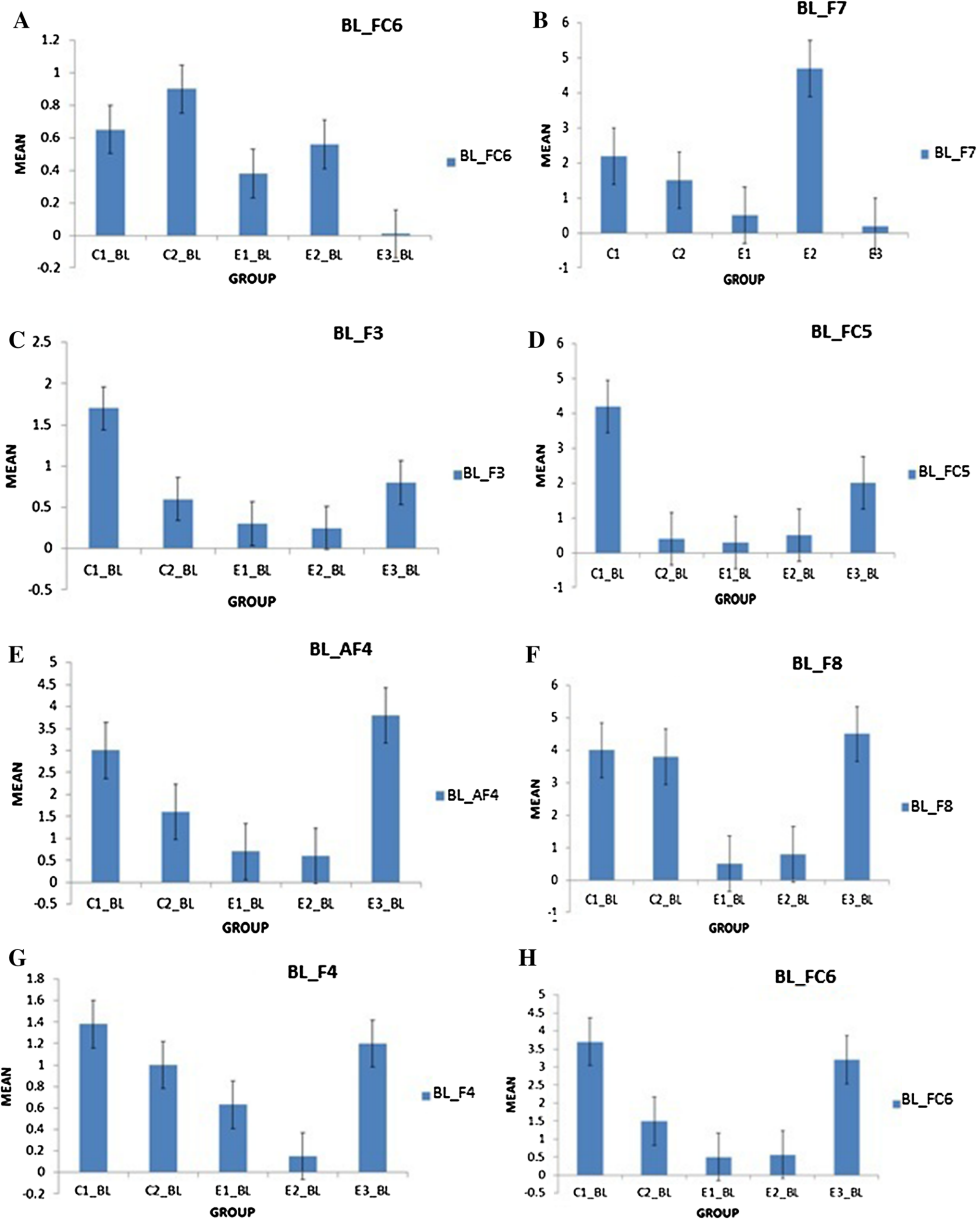
Beta energy (13–30 Hz) average plotted for baseline (BL) condition between groups as to demonstrate energy distribution between pre- and post-conditions to highlight effect of SKY with standard error as error bar. Units were displayed as a magnitude of 1*100000. *BL_AF3, BL_F7, BL_F3, BL_FC5, BL_AF4, BL_F8, BL_F4, BL_FC6* represented mean energy variance for baseline condition for EEG channels AF3, F7, F3, FC5, AF4, F8, F4, FC6, respectively

**Fig. 9:**
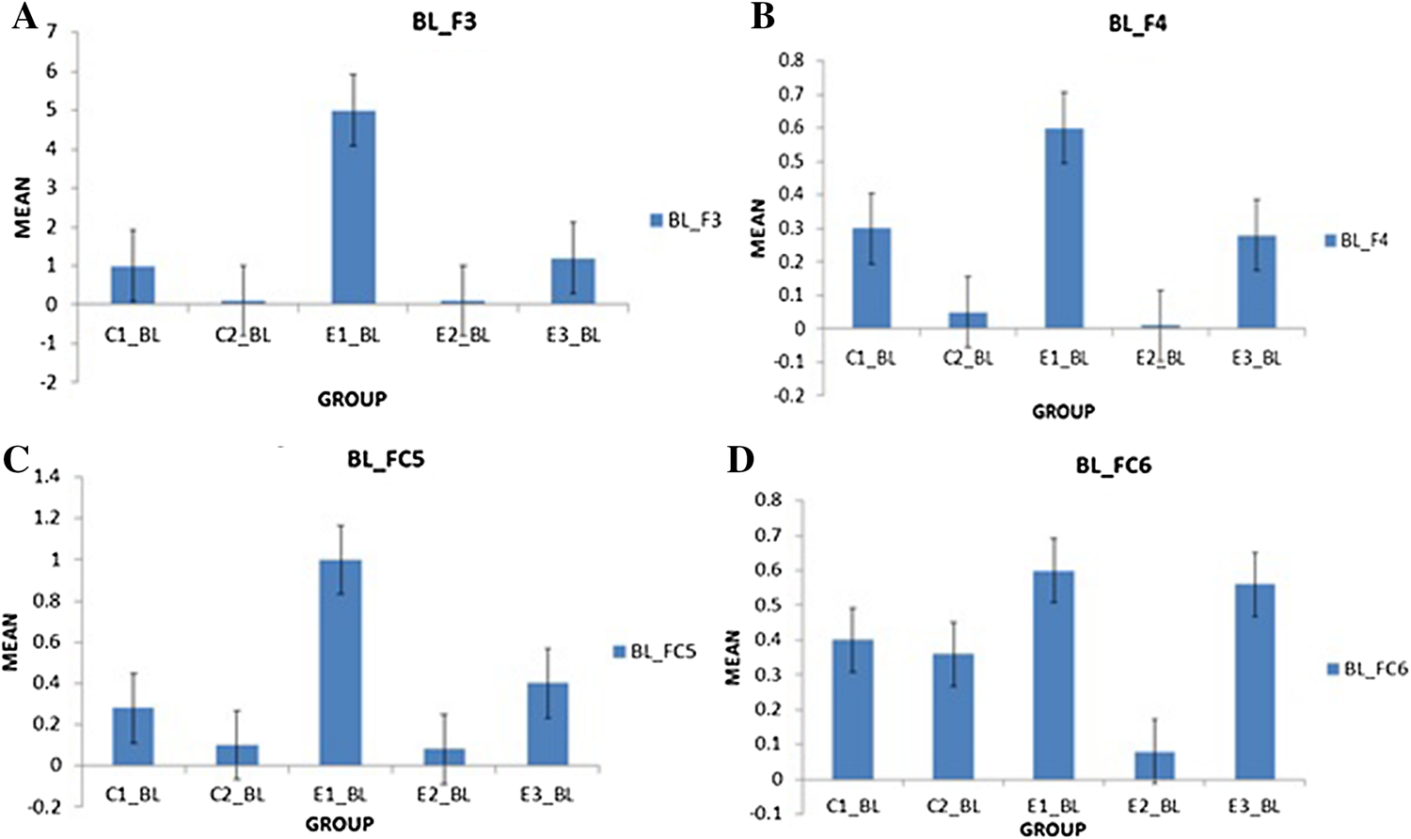
Gamma energy (30–45 Hz) average plotted for baseline (BL) condition between groups as to demonstrate energy distribution between pre- and post-conditions to highlight effect of SKY with standard error as error bar. Units were displayed as a magnitude of 1*100000 in vertical axis. *BL_F3, BL_FC5, BL_F4, BL_FC6* represented mean energy variance for baseline condition for EEG channels F3, FC5, F4, FC6, respectively

**Fig. 10:**
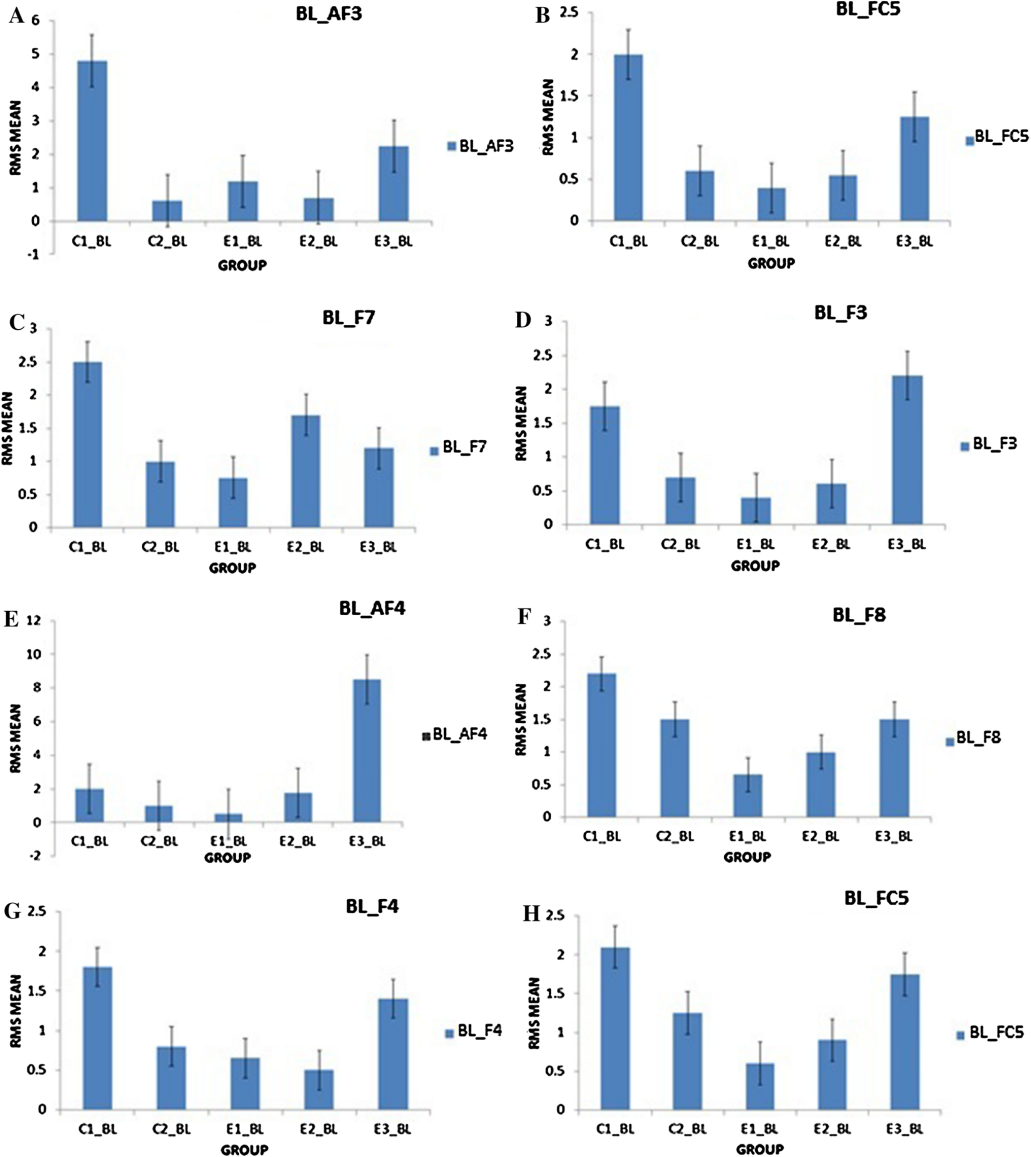
Alpha RMS average plotted for baseline (BL) condition between groups as to demonstrate energy distribution between pre- and post-conditions to highlight effect of SKY with standard error as *error bar*. *BL_AF3, BL_F7, BL_F3, BL_FC5, BL_AF4, BL_F8, BL_F4, BL FC6* represented mean RMS values for baseline condition for EEG channels AF3, F7, F3, FC5, AF4, F8, F4, FC6, respectively

**Fig. 11:**
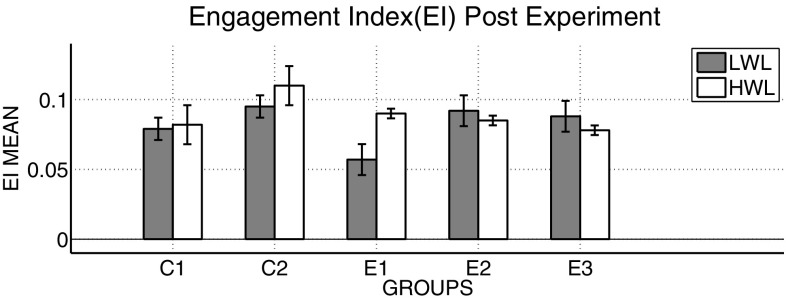
Engagement index (EI) values had been displayed in comparison for both groups

**Fig. 12:**
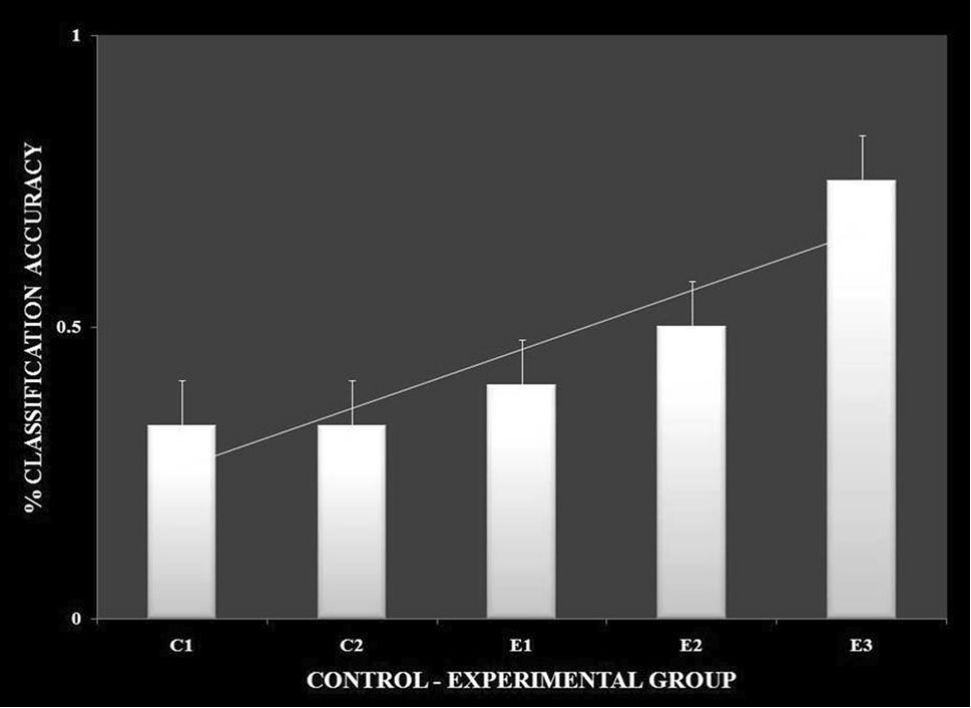
*Bar plots* showing percentage classification accuracy for LWL and HWL conditions between groups using support vector machine (SVM) algorithm

**Fig. 13:**
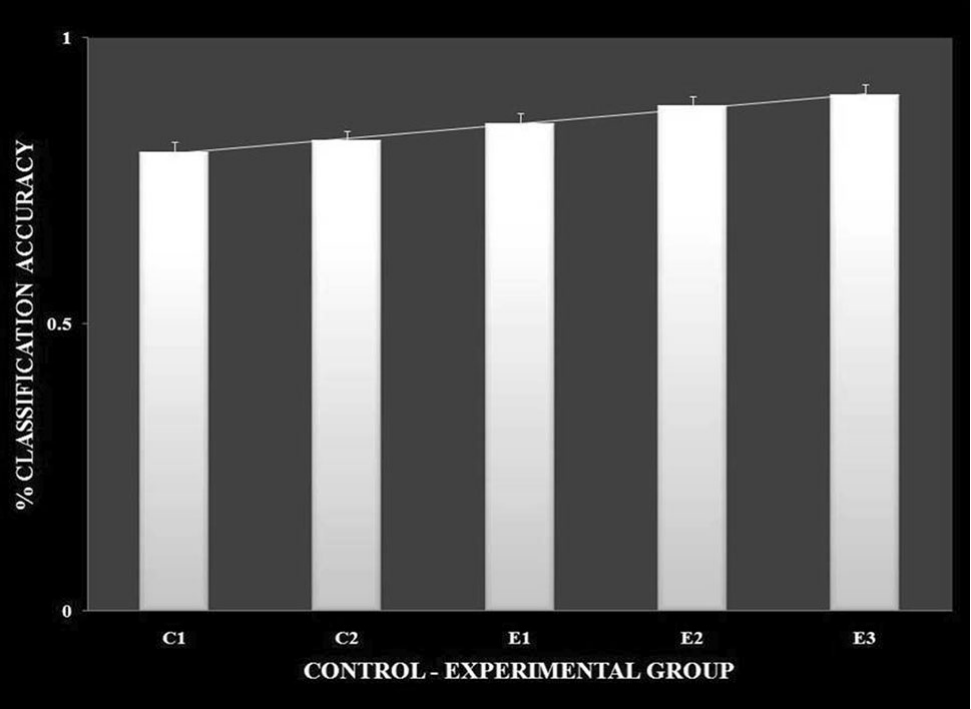
*Bar plots* showing percentage classification accuracy for LWL and HWL conditions between groups using neural network(NN) classifiers

It was observed in the experimental group that there was a significant reduction in subjective score for LWL (*F* = 51.01, *p* < 0.05) and HWL (*F* = 56.783, *p* < 0.05) while in the control group there was a reduction in score for LWL (*F* = 0.608, *p* = 0.44) and an increase in score for HWL (*F* = 3.514, *p* = 0.075) as shown in Fig. 1. For the tracking task, only RMSD-C score was taken which was the root mean square deviation from the center point in pixel units. In control group, RMSD value decreased for both LWL (*F* = 0. 341, *p* = 0. 559) and HWL (*F* = 6. 55, *p* = 0. 011). Similarly, in experimental group also, the RMSD value decreased for both LWL (*F* = 0.753, *p* = 0.386) and HWL (*F* = 10. 87, *p* = 0. 01). Within the pre- and post-groups, the RMSD value decreased for HWL, just because the tracking task got automated when the subjects cross a certain threshold level of workload and the stress level increased in the task, as shown in Fig. 2. For System Monitoring task, response time was taken as a performance variable for this task. There was a certain time duration in which the subject needs to give a response and if he failed to do so, then those responses were counted in negative value. The responses that were not answered taken as invalid cases, so that there was only response time of valid cases. In C2, number of invalid cases decreased. In both C1 and C2, the valid case increased for HWL and the response time increased in C2 for the valid cases (LWL: *F* = 3.77, *p* = 0.06; HWL: *F* = 0.56, *p* = 0.45). In experimental group, valid cases increased for HWL and LWL in both pre- and post-conditions and the response time decreased for E2 (LWL: *F* = 1.13, *p* = 0.29 and HWL: *F* = 0.31, *p* = 0.57) (Fig. 3). In addition to this, there was a decrement in invalid cases for E2. HRV was mostly correlated with the stress factor; therefore, SVI (SVI, ratio of LF to HF), NN-50, and mean RR interval were considered to identify the most significant effect of SKY on stress (Figs. 4, 5, 6). In experimental group, there was significant reduction in SVI (*F* = 5.33, *p* = 0.028) during rest condition as illustrated in Table 1. Although EEG signals were acquired from a 14-channel device, only eight channels i.e., F3, F4, AF3, AF4, F7, F8, FC5, and FC6 were utilized to assess the effect of SKY on workload more accurately as these locations were directly linked to the workload variance [24–26]. Comparison was made in pre- and post-control and experimental groups for theta (4–8 Hz), alpha (8–13 Hz), beta (13–30 Hz), and gamma (30–45 Hz) band. In rest condition, alpha and beta energy decreased for control group while they increased in experimental group. This was the most significant post-effect of SKY (Figs. 7, 8). Although gamma energy decreased for E2, it increased in E3 (lesser than E1) after SKY intervention. Similar reduction was visible in C2 (Fig. 9). There was an increase in synchronization in alpha sub-band during rest condition as depicted in Fig. 10. Result indicated decrease in EI when subject was in HWL after sky intervention (Fig. 11). There was a significant increase in EI for C2 in HWL as predicted by one-way ANOVA and is depicted in Table 2. Extracted EI feature was the most salient feature out of energy and RMS, and therefore, it was taken as an input in classifiers. Also, EI had complex relationship with the workload levels [18, 26]. Classification of the extracted feature (EI) was done in order to check the integrity. In this technique, total datasets (EI features) were randomly distributed into three datasets, one was used for training (20 %), second was used for validation (15 %) and remaining one was used for testing (65 %). Classification was made into two levels of workload i.e., LWL and HWL using technique SVM (Fig. 12) and ANN (Fig. 13).

**Table 1:** One way ANOVA showed within the group differences for all three conditions i.e. baseline (BL), low workload (LWL), and high workload (HWL) for all three ECG variables, mean of R interval (RR mean), sympathovagal balance index(SVI), and pNN50

Pre–post difference in group	Condition in experiment	*F*-values for ECG variables					
		RR mean		SVI		pNN50	
Control group	BL	1.12		2.29		3.23	
	LWL	0.12		2.46		1.39	
	HWL	0.46		2.46		1.29	
Experimental group	BL	0.69		5.34*		0.08	
	LWL	0.29		3.78		0.19	
	HWL	1.08		0.56		0.02	

* Showed significance at *p* < 0.05

**Table 2:** EEG channels variation between pre and post condition in groups had shown. Three conditions; Baseline(BL),Low Workload (LWL),High Workload(HWL) were compared between pre and post time period of Pranayama using one-way Anova

Groups	Condition in experiment	EEG channels (*F*-value)							
		AF3	F7	F3	FC5	FC6	F4	F8	AF4
1. Control pre–post	BL	5.31*	11.72*	4.71*	17.71**	8.17*	1.07	4.93*	5.31*
	LWL	7.59*	7.43*	12.58*	17.41**	12.91*	1.29	16.48**	7.59*
	HWL	5.57*	10.60*	16.64**	5.27*	4.28	1.32	9.31*	5.56*
2. Experimental pre–post	BL	27.51**	10.60*	19.26**	9.65*	17.96**	5.59*	12.77*	11.22*
	LWL	8.91*	5.30*	6.98*	2.93	8.92*	2.58	9.58*	3.63
	HWL	24.93**	8.19*	2.62	12.32*	1.96	1.83	3.79	4.55*

Three conditions; baseline(BL), low workload (LWL), high workload (HWL) were compared between pre- and post-time periods of Pranayama using one-way Anova

* Signifies less significant at *p* = 0.05

** Signifies more significant at *p* = 0.05

## Discussion

The experimentation was carried out to answer the question regarding how SKY can be helpful for increasing workload capacity by regulating stress generated due to task. Although it is scientifically known that SKY shows a positive effect in enhancing attention and vigilance [1] and reducing stress and anxiety [2], there is no empirical method which can correlate it with workload. In this pilot study, we tried to establish the role of SKY in enhancing workload tolerance capacity through regulating stress. MATB-II was used for workload assessment since it involved multitasking and divided attention tasks [7, 27]. LWL and HWL were easily achieved in test module by modifying the script in MATB-II. Performance was the explicitly measurable quantity of the experiment which linked SKY with workload. It was assessed by objective scoring obtained in system monitoring and tracking task while the rest of the tasks in MATB were used as a distraction for the participant. Subjective feedback was taken using NASA-TLX from the participants so that their own feeling towards different levels of workload could be assessed [28]. SKY had an immediate effect on the brain and the heart and in order to quantitatively assess them, the EEG and ECG recordings were carried out. Results showed that SKY had improved the reaction time, reduced the error, and increased the subjective score. SKY had a positive effect on cognitive flexibility which was reflected in the reduced RT. It made attentional switching easy; henceforth, it reduced latency period. In experimental group, there was a significant reduction in score for LWL and HWL while in the control group, score reduction was observed for LWL only. In this context, SKY had increased the capacity for WL tolerance; therefore, improvement in subjective response was observed. ECG was also used for workload discrimination and there was a direct link between HRV and Mental workload [19–21]. In E2 BL, a significant reduction in SVI was observed which indicated the effect of SKY on heart.

From an EEG perspective, most of the results were compared during rest condition i.e., BL among groups. It was done because the analysis of EEG signals showed an effect of SKY in baseline condition in E2 and E3 groups, but failed to effectively differentiate between LWL and HWL. The reason for selecting frontal and fronto-central channels was that these channels act directly as an index of information processing for both SKY as well as workload [24–26]. Alpha and beta energy blocking was observed from frontal and fronto-central channels for rest condition in C2 while the opposite was observed in E2. Henceforth, it was deduced that SKY enhanced alpha activity in rest condition that promotes blissful stage [1, 29]. Also, it was inversely related to stimulus discriminability [30] and attentional suppression of distracting information [31]. Alpha energy decreased in LWL due to left hemispheric activation during task. For control group, alpha energy was suppressed for all channels but for experimental group it was found only for AF3, F3, and F7 channels as these channels were involved in categorization tasks by using action knowledge [32]. Alpha RMS and beta energy increased for E2, and it was due to the increase in hemispheric synchronization. Frontal asymmetry was observed in E2 for LWL condition. A reduction in beta energy was observed in channels FC5 and FC6 for E2 in HWL condition. Beta energy increased when there was a reduction in target detection accuracy [33] or in speed of visual and sensorimotor processing [34]. Gamma energy decreased for both groups after 30 days but increased in E3 after 90 days. It could be estimated that gamma energy increased due to the reorganization of cortical oscillations modulating cognitive domain reorganization. Also, it was reported that gamma band activity increased in experienced practitioners [35]. Some researchers observed that beta and gamma oscillations accompany alpha increases which might be the answer of increased beta and gamma activities [36] after SKY during rest condition. To discriminate workload efficiently in the given task, there was a need to include nonlinear classifier with best EEG feature. To correlate EEG feature with workload was a challenge for us as the segregation of neural activity at particular channel was not possible. However, we tried it by identifying most prominent feature with the help of classifier. SVM and ANN were the most popular classifiers [11, 24] which were used in this pilot study. Engagement Index was the most prominent feature as determined using one-way ANOVA. When both classifiers run on EI, ANN proved to be a better technique than SVM. Also accuracy increased further after SKY, as it might indicate an improvement in EI with SKY.

## Conclusion

The present work is carried out to prove that Pranayama has an effect in regulating stress which in turn is reflected in enhancing the workload capacity. Consequently, a relationship is established between SKY and workload with the help of EEG and ECG signals. Differences are clearly taken into account during rest condition for both groups but discrimination between LWL and HWL is not done empirically. There is an increase in SVI component in the control group along with a decrease in alpha for baseline condition. Frontal asymmetry is found for alpha activity for E2 during LWL. Nevertheless, performance was improved for experimental group since their ability for handling stress had increased which increased their workload capacity. Subjects, who had already experienced SKY, were more content with their life and willing to include SKY in their daily routine for lifetime. It has been reflected in their increase in subjective scores. Future work will intrigue the fact that how long does the effect of SKY remain in case the subject has left practicing it. Henceforth, it will also be taken into consideration for a thorough investigation of its effect while replicating real-life situation that can produce mental workload so that Pranayama effect can easily be spotted.
